# Risk factors for penile prosthesis infection: An umbrella review and meta-analysis

**DOI:** 10.1080/2090598X.2023.2242204

**Published:** 2023-07-29

**Authors:** Konstantin Menshchikov, Mikhail Menshchikov, Dmitry Yurasov, Anton Artamonov

**Affiliations:** a The Russian Medical Academy for Continuing Professional Education (RMANPO), Moscow, Russia; b Institute of Biomedical Problems of the Russian Academy of Sciences, Moscow, Russia

**Keywords:** penile prosthesis infection, diabetes mellitus, glycated hemoglobin (HbA1c), antibiotic-coated, type of penile prosthesis

## Abstract

**Background:**

As available data on implantation-related infections is contradictory, the aim was to identify the predictors of penile prosthesis infection.

**Methods:**

We performed an umbrella review and meta-analysis including systematic reviews with extractable data. Literature search was done in two databases: PubMed and Google Scholar. The participants were males with erectile dysfunction regardless of etiology who underwent penile implant surgery. Using a standardized form, three trained researchers reviewed each reference (systematic review) by title and abstract. The meta-analysis was performed using Review Manager 5.4.1 (RevMan® 5.4.1).

**Results:**

A total of 78 systematic reviews were identified with the search strategies. Of these, 35 duplicates were removed. Thirty-seven full-text reviews were then excluded after revision. Six systematic reviews with a total of 271,226 patients (156,553 patients in the study group and 114,673 patients in the control group) were included in the meta-analysis. The analysis identified various predictors of adverse outcomes (infection). Among them were glycated hemoglobin (HbA1c) and different characteristics of penile implants.

**Conclusions:**

The systematic review and meta-analysis revealed significant risk factors/predictors of penile prosthesis infection: glycated hemoglobin levels; reoperation, and two predictors associated with the type of penile prosthesis. The weighted mean HbA1c levels of patients with and without infections were 8.37% and 7.17% respectively. The OR was as follows: first surgery/revision OR 0.36 (95% CI 0.29–0.45); antibiotic-coated/non-coated prosthesis OR 0.47 (95% CI 0.31–0.72); malleable/inflatable prosthesis OR 3.51 (95% CI 1.41–8.74).

## Introduction

The number of penile implant surgeries has been growing steadily from year to year [1]. At the same time, complications such as mechanical malfunction, infection, and erosion still occur. The proportion of complications depends on many factors (the condition of the patient, primary implantation or reimplantation, type of prosthesis, etc.). The most significant complication is infection [1]. Complications such as infection are closely related to risk factors such as diabetes mellitus (DM) and reimplantation. At the same time, diabetes mellitus (DM) cases have also been on the rise [2] and approximately 50% of these patients (with diabetes) have some degree of erectile dysfunction [1]. According to the forecasts presented in [2] patients with type 2 diabetes will increase by about 4 times by 2050. Thus, we can predict the need for several million implantations per year of penile prostheses by 2050 and these predictions only apply to diabetic patients. Patients with long-term DM are at an increased risk of erectile dysfunction, and penile implant surgery for such patients is associated with higher infection rates [3] because of leucocytes dysfunction and microangiopathy [1]. On the other hand, prevention strategies and monitoring of patients with DM are constantly improving [4], so we hope that the risks of DM-associated infection will decline. Because of the importance of diabetes as a risk factor for both erectile dysfunction and prosthetic infection, we focused on the impact of diabetes and associated glycated hemoglobin (HbA1c) levels on infection risk after implantation. But despite the fact that many authors [1,3,5–8] agree to the impact of diabetes on the occurrence of prosthetic infection, there are studies that deny the predictive role of HbA1c levels [5–7]. The controversy surrounding the predictive role of HbA1c levels justify conducting this umbrella review and meta-analysis.

**Figure 1.**
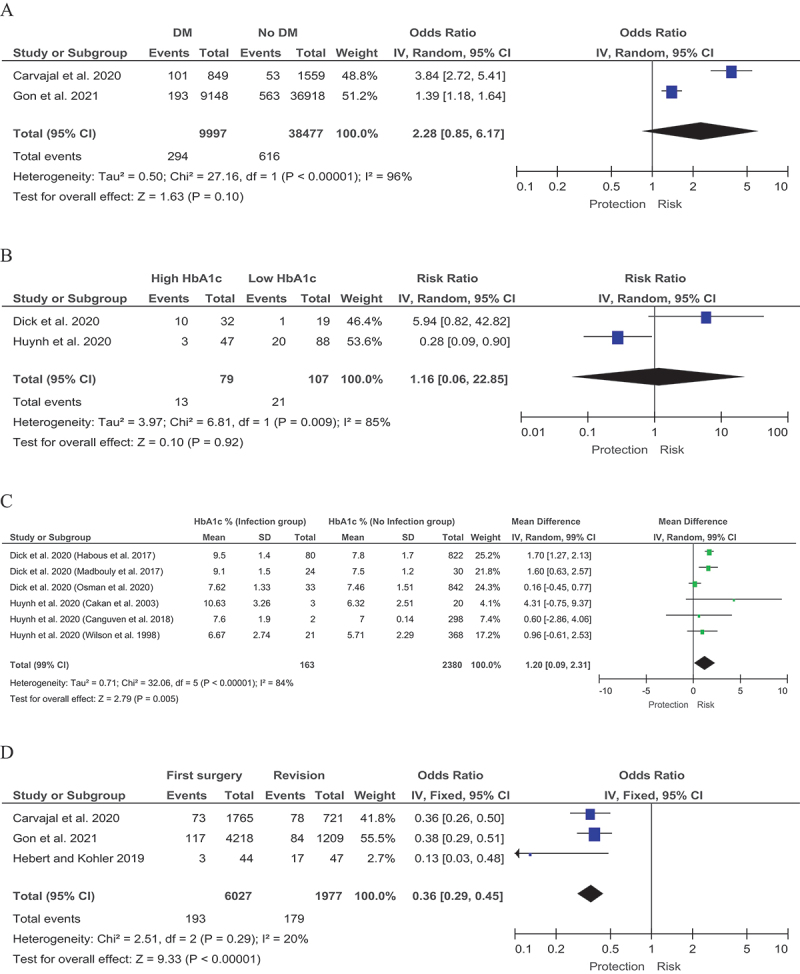
Forest plot for associated risk factors. A Diabetes mellitus. B Glycated hemoglobin (HbA1c) levels. C Difference in average glycated hemoglobin (HbA1c) levels. D Revision. E The “no touch” technique. F Antimicrobial coating. G Type of penile prosthesis.

**Figure continue.**
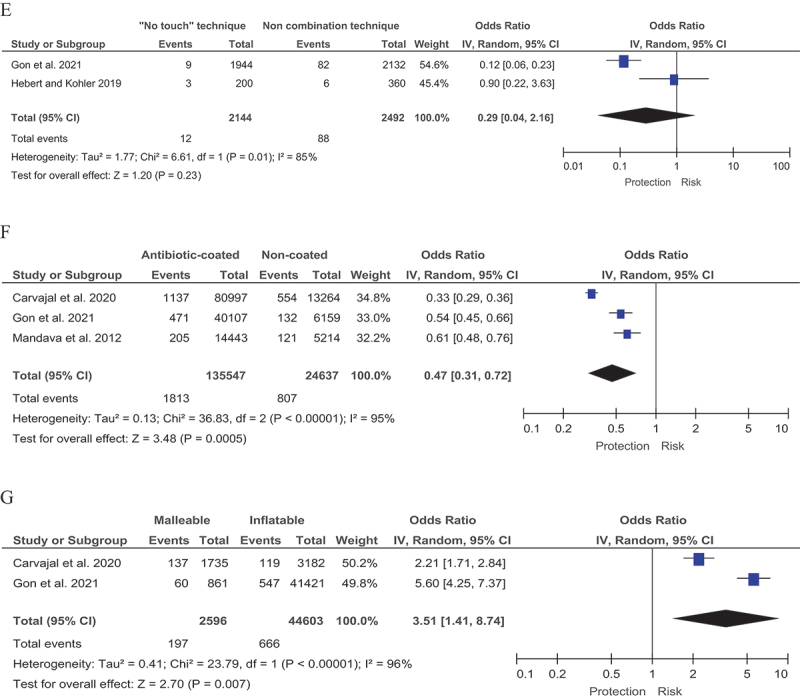

In addition to diabetes, there are other risk factors for prosthesis infection that can be divided into several types: risks associated with the patient’s condition; risks associated with the type of implant and risks associated with the procedure [1,3,5–8]. Urological surgeons must continue to strive for reduction of risks of infection during implantation. Infection leads to long-term treatment, reoperation and subsequent long rehabilitation. These challenges put a heavy burden on the patient, incur considerable material costs and affect the patient’s physical and mental well-being. One way of reducing these risks is careful planning prior to surgery and taking predictors into account. The goal of this work was to identify the most significant risk factors for penile prosthesis infection, such as glycated hemoglobin levels, type of prosthesis, and risks associated with the implantation procedure.

Assessment and accounting of the risks of infection of the penile prosthesis is possible on the basis of a large amount of statistical data. For this review, we extracted a large amount of data sufficient to assess the risks associated with reimplantation and non antibiotic-coating and DM. An umbrella review and meta-analysis of these risk factors is justified as it opens the way for subgroup analyzes (such as analysis of HbA1c levels in the group of patients with diabetes mellitus).

## Methods

We sought to identify the predictors of penile prosthesis infection according to the recommendations of the PRISMA Statement (preferred reporting items for systematic reviews and meta-analyses). The PROSPERO registration number is CRD42023418279. Search depth from 2012 to 2023. The search terms applied were as follows: (penile prosthesis infection [Title/Abstract] OR risk factors infection penile prosthesis implantation [Title/Abstract])

## Inclusion criteria

### Study design

Only systematic reviews were included.

### Participants

Male patients with erectile dysfunction regardless of the etiology and who received a penile implant.

### Comparisons

Infection rates between the study group and control group were compared.

### Primary outcome

Predictors of infection after penile prosthesis implantation including local and systemic infections.

### Secondary outcome

Predictors of secondary infection leading to reoperation.

### Timing

We limited the time of onset of an adverse outcome (infection after prosthesis implantation) to no more than one year. Infection usually develops within one month after surgery, but not more than one year after surgery. Cases of infection other than infection after surgery were excluded.

## Exclusion criteria

Data from systematic reviews utilizing cohorts already analyzed in another review was excluded. Case reports or papers with data non-eligible for meta-analysis were not considered. Transgender patients were not included.

## Data collection

Three trained researchers used a standardized form to independently extract the following information from each systematic review: authors’ names, title, year of publication, study period, inclusion and exclusion criteria, number of patients included, losses to follow up and number of favorable and unfavorable outcomes.

Disagreements were resolved by consensus and where agreement could not be reached, the fourth expert resolved the conflict.

### Information sources

Literature search was performed in two databases: PubMed and Google Scholar. We checked all data extracted from systematic reviews and double-checked the original source.

## Data analysis/synthesis

The statistical analysis was performed using RevMan® 5.4.1. For categorical outcomes we reported odds ratios (OR) with 95% confidence intervals according to the type of variables. We pooled the information with random-effects meta-analysis according to the heterogeneity expected (where possible). The results are shown in forest plots of the estimated effects of the included studies with a 95% confidence interval (95% CI). Heterogeneity was evaluated with the I [2] test. The I [2] heterogeneity index was then used to interpret the results (0–40% - slight heterogeneity, 30–60% - moderate heterogeneity, 50–90% - significant heterogeneity 75–100% - high heterogeneity). The χ^2^ criterion was used to obtain p-values. A p-value of 0.1 was selected as the threshold for statistically significant heterogeneity.

### Sensitivity analysis

A sensitivity analysis extracting weighted data from included studies was performed.

Effect or random effects were estimated to identify differences.

### Analysis of subgroups or subsets

Predictor type (associated with the patient or prosthesis type).

## Results

A total of 78 reviews were identified with the search strategies. Of these, 35 duplicates were removed. Thirty-seven full-text reviews were then excluded after revision. Six systematic reviews matched the inclusion criteria and were found suitable for data extraction: Gon et al. 2021 [1], Carvajal et al. 2020 [3], Dick et al. 2020 [5], Hebert and Kohler 2019 [6], Huynh et al. 2020 [7] and Mandava et al. 2012 [8]. A PRISMA flow diagram which reflects database searches is given in the supplementary material section.

## Included studies

A total of 271,226 patients were included in the meta-analysis (156,553 patients in the study group and 114,673 patients in the control group).

## Predictors related to the patient

### Diabetes mellitus (DM)

Two systematic reviews by Gon et al. [1] and Carvajal et al. [3] with a total of 48,474 patients explored the risks associated with DM. In these reviews, 9,997 patients had diabetes mellitus and 38,477 did not. Complication rates after implantation were 2.94% (294/9997) in the DM group and 1.6% (616/38,477) in patients without DM. An association between DM and risk of infection was found: OR 2.28 (95% CI 0.85–6.17) I [2] = 96% (Fig. 1A). However, the difference was not statistically significant (p-values = 0.1).

## Glycated hemoglobin (HbA1c) levels

Glycated hemoglobin (HbA1c) was selected as a potential predictor of infection in patients with DM. In this approach, we evaluated a variant predictor based on the classification of HbA1c levels: low HbA1c and high HbA1c. Two studies by Dick et al. [5] and Huynh et al. [7] were included with a small total number of patients of 186. In the group of patients with high HbA1c levels, 16.46% (13/79) developed infections, in contrast to the low HbA1c group where infection rates reached 19.63% (21/107). However, the meta-analysis showed a slightly higher risk of infection in patients with high HbA1c levels compared to those with low HbA1c: OR 1.16 (95% CI 0.06–22.85) I [2] = 96% (p-values = 0.92) (Fig. 1B).

## Difference in average glycated hemoglobin (HbA1c) levels

To address the problem of uncertainty in HbA1c levels described above, a meta-analysis of data on mean HbA1c levels in patients with and without complications (infections) after implantation was performed (Fig. 1C). This meta-analysis combined the patients in papers by Dick et al. [5] and Huynh et al. [7]. The resulting groups with and without infection after implantation numbered 163 and 2,380 patients respectively. Given that some sources reported fasting blood glucose measurements, we used the model proposed by Xu Y et al. [4] to convert fasting glucose levels to HbA1c levels, thereby increasing the amount of data for meta-analysis. The difference in mean HbA1c levels was 1.20 (95% CI 0.09–2.31) I [2] = 84%, (p-values = 0.005). The weighted mean HbA1c level of patients with infection was 8.37% and that of patients without infection 7.17%.

## Predictors related to the procedure

One important predictor of a possible adverse outcome (infection) is reoperation. Three systematic reviews by Gon et al. [1], Carvajal et al. [3] and Hebert and Kohler [6] were used to assess the effects of repeat surgery. The total number of patients who underwent surgery was 6,027 with a complication rate of 3.20% (193/6,027). The total number of patients who underwent reoperation was 1,977, with a complication rate of 9.03% (179/1,977); OR 0.36 (95% CI 0.29–0.45) I [2] = 20% (p-values <0.00001) (Fig. 1D).

## The “no touch” technique

The ‘no touch’ technique can be used as an option to reduce the risk of infection. In order to investigate the impact of the ‘no touch’ technique, we conducted studies that included two systematic reviews by Gon et al. [1] and Hebert and Kohler [6]. The total number of patients operated on using the ‘no touch’ technique was 2,144 with a complication rate of 0.56% (12/2,144), while 2,492 patients were operated on initially without the combined technique with 3.53% (88/2,492) developing complications. Note that the complication rate agrees well with the after primary surgery in the previous section. In terms of efficacy, the ‘no touch’ technique was more effective than the non-combined surgical technique as shown by an OR of 0.29 (95% CI 0.04–2.16) I [2] = 85% (p-values = 0.23) (Fig. 1E).

## Predictors related to the implant

### Antimicrobial coating

An obvious predictor is antimicrobial coating of the penile implant surface. To test this predictor for statistical significance we used three systematic reviews by Gon et al. [1], Carvajal et al. [3] and Mandava et al. [8]. The number of surgeries with antibiotic-coated prostheses was 135,547 with a complication rate of 1.34% (1,813/135,547), in contrast to the cases with non-antibiotic-coated prostheses with a complication rate of 3.28% (807/24,637). The difference was statistically significant: OR 0.47 (95% CI 0.31–0.72) I [2] = 95% (p-values = 0.0005) (Fig. 1F).

## Type of penile prosthesis

The last predictor to consider is the type of prosthesis. Malleable and inflatable prostheses were compared. The results were presented by two systematic reviews by Gon et al. [1] and Carvajal et al. [3]. In total, there were 2,596 patients in the malleable implant group with an infection rate of 7.56% (197/2,596), and 44,603 patients in the inflatable implant group with an infection rate of 1.49% (666/44,603). Statistical significance was high with OR of 3.51 (95% CI 1.41–8.74) I [2] = 96% (p-values = 0.007) (Fig. G1).

## Discussion

### Predictors related to the patient

One of the key findings is that diabetes itself is no longer such a critical factor in prosthetic infection. No statistically significant difference in implantation outcomes between the groups with and without diabetes has been found. We believe this is due to significant improvement in diabetes management and increased patient awareness of the dangers of diabetes mellitus and the importance of prevention and self-management strategies [4]. On the other hand, individuals with diabetes who fail to adequately control their condition remain at risk of infection. We concluded that a rough estimate of ‘high/low’ HbA1c levels is not a meaningful prognostic criterion due to the fact that the definition of ‘high’ or ‘low’ HbA1c varies from author to author. Moreover, normal HbA1c ranges depend on various factors, e.g. race, gender and age [4]. This could be explained by the fact that different publications describe different thresholds for HbA1c levels. Moreover, the number of patients in this paper is small. It is reasonable to state that there is no agreed upon threshold value but higher HBA1c levels are associated with higher risk of infection.

A quantitative analysis of the association between prosthesis infection and blood HbA1c levels showed significant statistical difference. The study design itself is, in our opinion, highly objective, as HbA1c levels were measured in patients with and without infection. Thus, HbA1c levels in patients with DM may be considered a reliable and valid predictor. We recommend informing patients with high HbA1c levels (above 8%) about preventive measures and discussing the risk of infection after implantation and suggest that surgeons consider postponing implantation until the patient’s glycated hemoglobin has returned to normal values. Furthermore, both the surgeon and the patient must take into account that the risk of infection after repeat surgery is approximately 3 times higher than following the initial intervention. For this reason, improving the chances of success of the initial operation is critical and requires effort on the part of physicians and patients.

## Predictors related to the procedure

The ‘no touch’ combination technique has been suggested as one of the options for increasing the chances of a favorable outcome more than threefold. However, our analysis showed that there are no statistically significant differences in the outcomes of surgery with and without the ‘no touch’ technique. Admittedly, the small number of patients and unequal distribution of the extracted data are significant limitations of our analysis, and we are aware of a high risk of bias regarding this part of the study. Hopefully, more data on the promising ‘no touch’ combination technique will be accumulated in the future.

## Predictors related to the implant

Antibacterial coating of the penile prosthesis has a significant impact on the surgical outcome. The risk of infection is more than halved with antibiotic-coated prostheses, and the difference is highly statistically significant. Coating of the penile prosthesis is a reliable predictor and can be used to decide on an implantation strategy. Where an antibiotic-coated prosthesis cannot be used, antibiotic therapy should be planned. Antimicrobial therapy is an important measure that must be addressed with an understanding of the types of bacterial infections that commonly occur following implant surgery. There is a need to structure the accumulated knowledge and develop models. We plan on developing this aspect further. The last important predictor is the type of prosthesis. We have analyzed two general prosthesis types: malleable and inflatable. These two types differ both cost- and function-wise. As there is a significant statistical difference between the surgical outcomes with these two prosthesis types, we found the largest (more than 3.5-fold) difference between the patient groups. This predictor will allow surgeons to significantly reduce the risk of infection in settings where no other means of risk reduction are available.

Despite the large number of patients (apparently the largest up to date), our research has certain limitations. First of all, the data covers a considerable time span of up to 40 years. Obviously, the quality of medical service, surgical techniques and prostheses has been improving annually. Nevertheless, this data coverage allowed us to select the most appropriate and reliable predictors of adverse implant outcomes.

## Conclusion

This large-scale meta-analysis revealed several significant predictors: glycated hemoglobin levels, reoperation and two predictors associated with the type of prosthesis. They will allow surgeons to manage the risks associated with infection after implantation.

## Supplementary Material

Supplemental Material
