# Supplementary material for: Risk factors for penile prosthesis infection: An umbrella review and meta-analysis
Source: Arab J Urol. 2023 Jul 29;22(2):96–101. doi: 10.1080/2090598X.2023.2242204 (PMC10929667; doi:10.1080/2090598X.2023.2242204)
Supplement: Supplemental Material [file TAJU_A_2242204_SM7065.docx]

**Identification of studies via databases**

**Identification**

Records identified from

Databases (n=780)

Records removed *before screening*:

Duplicate records removed

(n =35)

Records marked as ineligible by automation tools - were not reviews (n=702)

**Screening**

Records screened

(n= 43)

Records excluded

(n=37) - were not systematic reviews

**Included**

Studies included in review

(n= 6)

PRISMA flow diagram which included searches of databases
